# Atypical Kinetics and Albumin Effect of Glucuronidation of 5-*n*-Butyl-4-{4-[2-(1*H*-tetrazole-5- yl)-1*H*-pyrrol-1-yl]phenylmethyl}-2,4-dihydro-2-(2,6- dichlorophenyl)-3*H*-1,2,4-triazol-3-one, a Novel Nonpeptide Angiotensin Type 1 Receptor Antagonist, in Liver Microsomes and UDP-Glucuronosyl-transferase

**DOI:** 10.3390/molecules23030688

**Published:** 2018-03-19

**Authors:** Ying Peng, Xueyuan Zhang, Yinci Zhu, Hui Wu, Shiyin Gu, Qingqing Chang, Yi Zhou, Guangji Wang, Jianguo Sun

**Affiliations:** Key Lab of Drug Metabolism and Pharmacokinetics, State Key Laboratory of Natural Medicines, Pharmaceutical University China, Nanjing 210009, Jiangsu, China; 1020162518@cpu.edu.cn (Y.P.); xueyuanzh@yeah.net (X.Z.); zhuyc0812@163.com (Y.C.Z.); wuhui0922@hotmail.com (H.W.); gsy.163@163.com (S.G.); 15298377114@163.com (Q.C.); zhouyi-1018@163.com (Y.Z.)

**Keywords:** Ib glucuronidation, enzyme kinetics, BSA effect, liver microsomes, UGTs

## Abstract

Ib is a new nonpeptide AT1 receptor antagonist, which plays an active role in cardiovascular protection. Ib monoglucuronide has been identified as its main metabolite. A detailed study of Ib glucuronidation is important for predicting potential DDI. Besides, the elucidation of the “BSA effect” in Ib glucuronidation would make obtained kinetic parameters more predictive in IVIVE. “BSA effect” means that there is a significant change in in vitro kinetic parameters when generated from incubations performed in the presence of bovine serum albumin (BSA). Five UGTs (UGT1A3, UGT2B4, UGT2B7, UGT1A9 and UGT1A8) were identified that produced abundant Ib monoglucuronide, especially UGT1A3. We investigated Ib glucuronidation in liver microsomes from different species (rat, dog, human) and in five identified major human UGTs. Ib glucuronidation in liver microsomes and recombinant human UGTs all showed substrate inhibition kinetics. DLM showed the strongest affinity and activity, HLM showed the lowest affinity, and RLM showed the weakest activity. The addition of BSA did not alter the enzyme kinetics, but significantly altered enzyme kinetic parameters resulting in a reduction in *K*_m_ value and an increase in CL_int_ value. However, high concentrations of BSA could significantly attenuate this positive effect on enzyme affinity and activity, and the effect of BSA on the *V*_max_ of Ib glucuronidation was opposite in different enzyme sources. In conclusion, this study demonstrated the substrate inhibition kinetics of Ib glucuronidation in the liver metabolism and the effect of BSA on its kinetic parameters, in order to provide more accurate in vitro data for in vivo prediction.

## 1. Introduction

**Figure 1:**
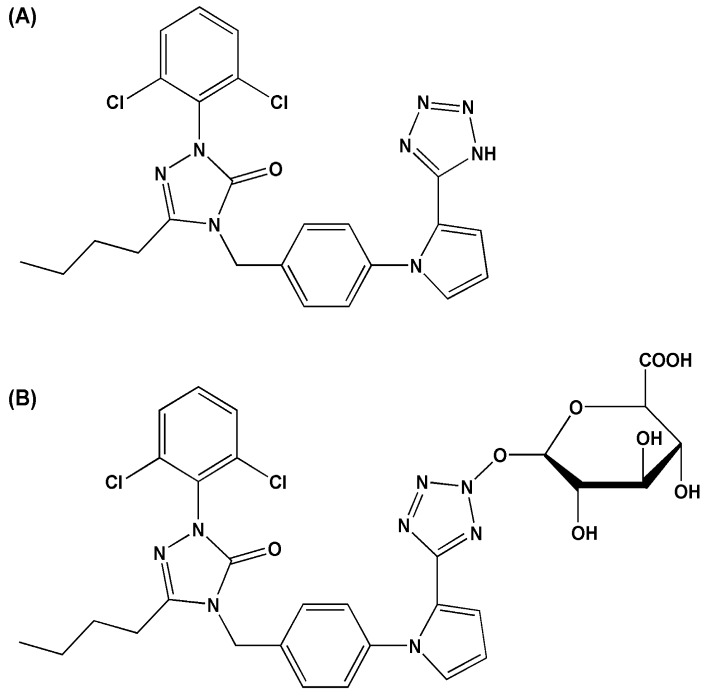
Structures of Ib (**A**) and Ib monoglucuronide (**B**).

Renin-angiotensin system plays a key role in blood pressure regulation and maintenance of body fluid balance. Angiotensin II is an important neurotransmitter in this system, mainly through AT1 receptor regulating the blood pressure. Ib (Figure 1A), a new nonpeptide AT1 receptor antagonist, was designed and synthesized by the Department of Medicinal Chemistry of China Pharmaceutical University based on the electronic biological isometric and flattening principle [1]. It has been proven to play an active role in cardiovascular protection, such as anti-hypertension [2], preventing cardiac hypertrophy [3], antagonizing vascular contraction [4], suppressing cell proliferation of vascular smooth muscle [5].

During early study by our laboratory [6], five main metabolites of Ib, including one dihydroxylated Ib, three monohydroxylated Ib and one Ib monoglucuronide, have been reported in rat bile. In this study, Ib monoglucuronide (Figure 1B) was found to be a common metabolite in liver microsomes of different species (mice, rat, dog, monkey and human). And its amount was abundant, accounting for more than 30% of all generated metabolites. Thus, further detailed study of Ib glucuronidation was of importance significance to elucidate Ib elimination. And the identification of UGT isoforms involved in Ib glucuronidation would help to predict potential drug-drug interactions and polymorphism-related interindividual variability.

However, the metabolic activity of the subcellular components used for metabolic studies (such as microsomes and recombinants) would be inhibited by the long chain unsaturated fatty acids released from membrane during the course of an incubation in vitro. And the addition of BSA could antagonize this inhibition effect and influence the enzyme activity of CYPs [7,8,9,10] and UGTs [11,12,13,14], yielding *K*_m_ and *V*_max_ estimates that are more representative for in vitro-in vivo extrapolation. Therefore, a series of BSA concentration would be adopted to adjust the kinetic data of Ib glucuronidation in liver microsomes and recombinant UGTs.

In summary, the main objective of this study is to characterize the glucuronidation of Ib in liver microsomes and recombinant UGTs, and to assess the BSA effect on the enzyme kinetics of Ib glucuronidation.

## 2. Results

### 2.1. Metabolites of Ib in Microsomes

Preliminary research by our laboratory has identified five metabolites of Ib in rat bile, including one dihydroxylated Ib, three monohydroxylated Ibs and one Ib monoglucuronide. In this study, we detected the metabolites of Ib generated in the liver microsomes from multiple species (HLM, MKLM, DLM, RLM, MLM) using similar analytical method as reported [6]. Finally, we found totally ten metabolites including four monohydroxylated Ibs, four dihydroxylated Ibs, one methylated Ib, and one Ib monoglucuronide. Among them, Ib monoglucuronide was existed in all samples and its amount was abundant. The percentage of Ib monoglucuronide in all investigated metabolites was about 31.8%, 56.5%, 30.6%, 44.5% and 43.5%, respectively, for HLM, MKLM, DLM, RLM and MLM. The glucuronidation might be the main metabolic pathway of Ib in the liver.

### 2.2. Screening by Recombinant UGTs

**Figure 2:**
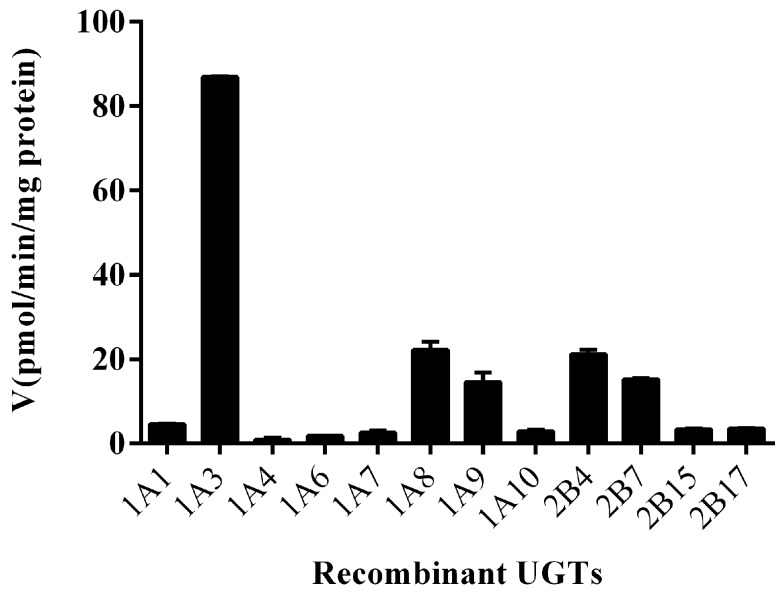
Formation of Ib glucuronide by recombinant human UGT isoforms. A total of 20 μM Ib was incubated with recombinant human UGT isoforms (UGT1A1, UGT1A3, UGT1A4, UGT1A6, UGT1A7, UGT1A8, UGT1A9, UGT1A10, UGT2B4, UGT2B7, UGT2B15, and UGT2B17) at a protein concentration of 0.1 mg/mL. Each bar is the mean ± S.D. of triplicate determinations.

As an initial screen, 12 commercially available recombinant UGTs were evaluated for their ability to metabolize Ib at a concentration of 20 μM. As shown in Figure 2, almost all recombinant UGTs could generated Ib monoglucuronide. Among them, five UGTs produced significant amount of Ib monoglucuronide, in the order of UGT1A3 > UGT1A8 > UGT2B4 > UGT2B7 > UGT1A9.

### 2.3. Binding of Ib to BSA

The unbound fractions (*f*_u_) of Ib at different concentrations determined in the presence of BSA are listed in Table 1. Binding of Ib to microsomal and supersome preparations was negligible (mean *f*_u_ range 0.91–1.08) in the absence of BSA. The binding of Ib to the enzyme source/BSA mixture was concentration-dependent by both the substrate concentration (10–2000 μM) and the BSA concentration (0.2–2%). As shown in Table 1, the *f*_u_ increased with the increase of Ib concentration, while the *f*_u_ decreased as the BSA concentration increased.

### 2.4. Ib Glucuronidation by Microsomes and BSA Effect 

**Figure 3:**
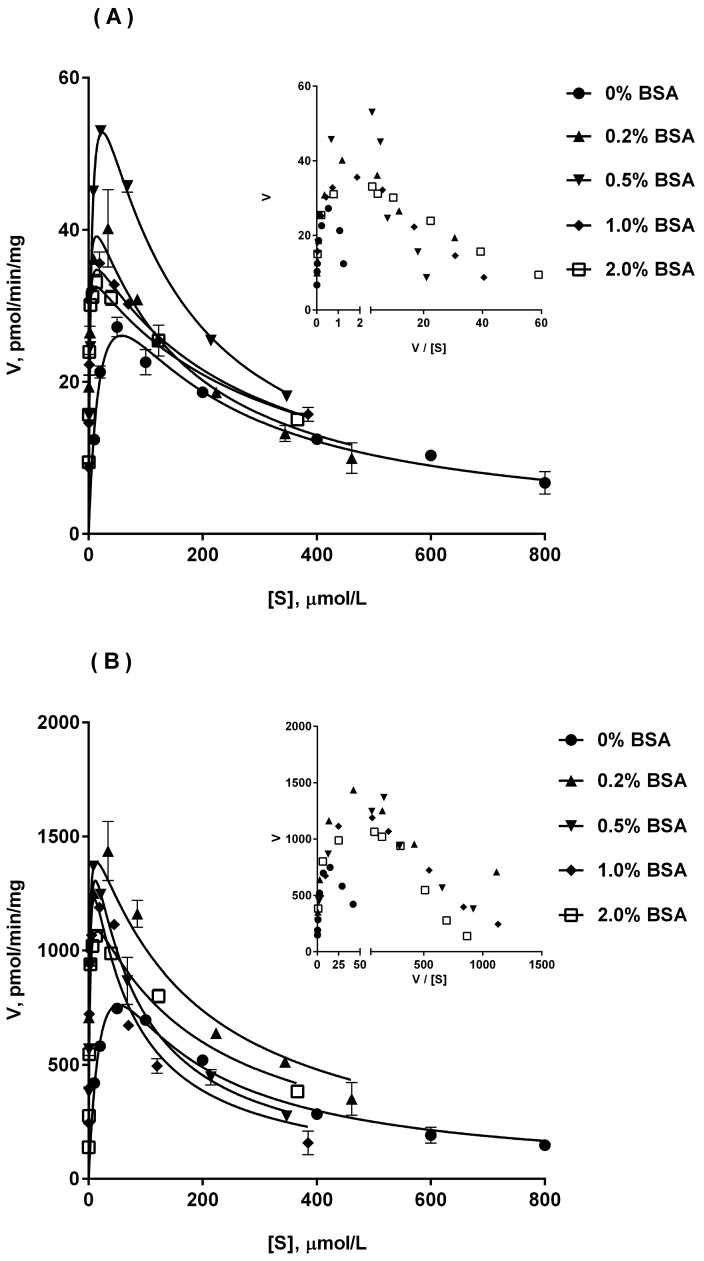

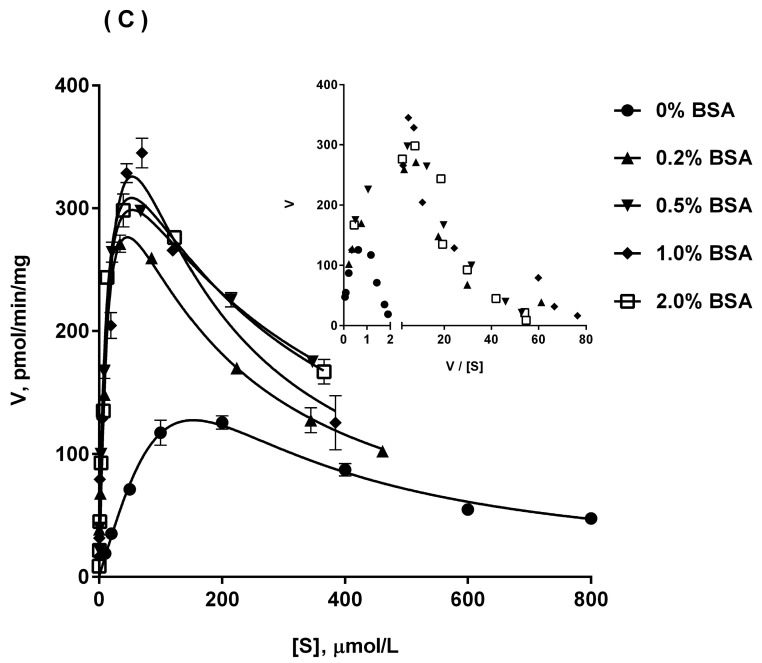
Atypical kinetic profiles for Ib glucuronidation in RLM (**A**), DLM (**B**) and HLM (**C**), in the absence and presence of BSA. Inset, Eadie-Hofstee plots for each of the profiles are shown. In the concentration of BSA at 0%, 0.2% and 0.5%, incubations were performed across 10 to 800 μM Ib concentration range. In the concentration of BSA at 1% and 2%, incubations were performed across 10 to 2000 μM Ib concentration range. The substrate concentration ([S]) in kinetic profiles refers to the unbound Ib concentration corrected by the free fraction (*f*_u_) of Ib. Each data point represents mean ± S.D. of triplicate determinations.

In this study, we investigated Ib glucuronidation in HLM, DLM and RLM, in the absence or presence of BSA (0.2–2%). As shown in Figure 3, we observed that Ib glucuronidation displayed substrate inhibition kinetics at higher concentrations on three liver microsomes, confirmed by Eadie-Hofstee plots, which showed a hook in the upper quadrant. Derived kinetic constants by fitting with unbound Ib concentrations are shown in Table 2. It was observed that Ib glucuronidation in liver microsomes had significant species differences. For the metabolic capacity, the relationship between the three is DLM > HLM > RLM, by comparing the *V*_max_ and CL_int_ values. For the metabolic affinity, the relationship between the three is DLM > RLM > HLM, while the highest *K*_m_ value corresponding to the weakest affinity.

The addition of BSA did not alter the enzyme kinetics of Ib glucuronidation in liver microsomes. However, the addition of BSA resulted in an order of magnitude improvement in CL_int_ values for all investigated microsomes with a sharp reduction in *K*_m_ values and an increase in *V*_max_ values. Among them, BSA could result in a reduction of approximately 90.2%, 84.9% and 85.5% on Km values for HLM, DLM and RLM, respectively, without dependent on BSA concentrations. But, the increase on *V*_max_ values varied with BSA concentration. With the increase of BSA concentration from 0.2% to 2%, the growth rate of *V*_max_ in RLM incubation first increased and then decreased, and 0.5% BSA corresponding to the highest *V*_max_ value. In the incubation with DLM, the growth rate of *V*_max_ decreased from about 92.4% to 32.8% with the increase of BSA concentration from 0.2% to 2%. For HLM, the effect of BSA on *V*_max_ was similar to that of RLM, but the effect was not significant and the highest *V*_max_ value was corresponding to 1% BSA. The results showed that too high BSA concentration like 2% would weaken the positive effects of BSA itself.

### 2.5. Ib Glucuronidation by Recombinant UGTs and BSA Effect 

**Figure 4:**
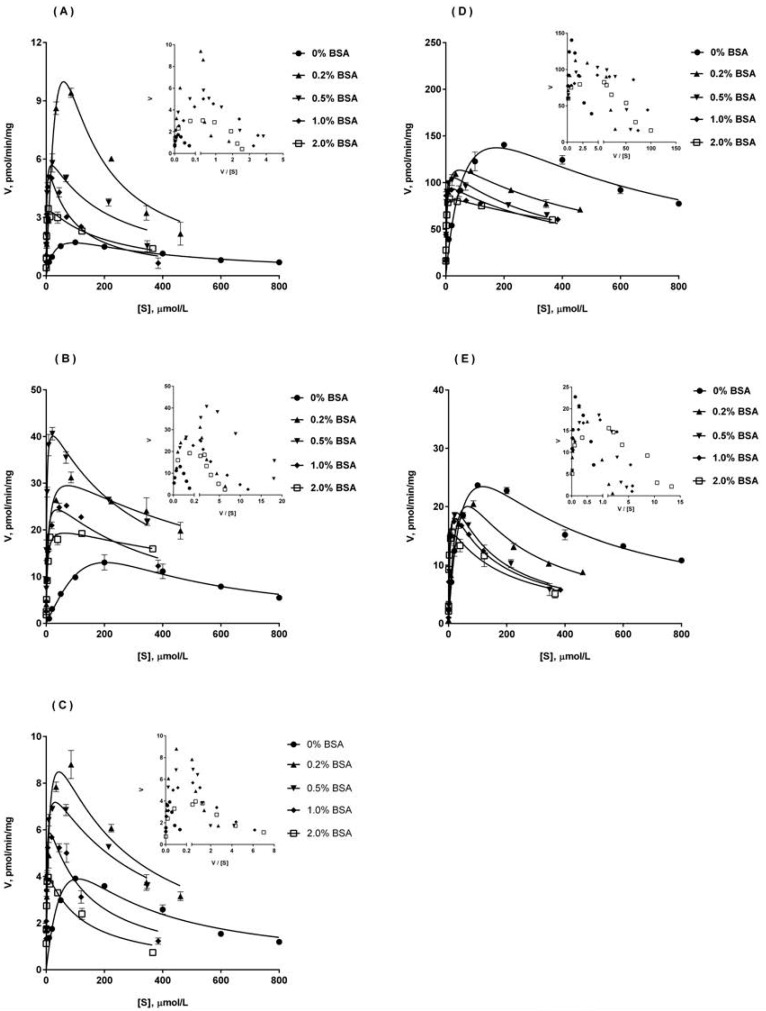
Atypical kinetic profiles for Ib glucuronidation in UGT1A9 (**A**), UGT2B4 (**B**), UGT2B7 (**C**), UGT1A3 (**D**) and UGT1A8 (**E**), in the absence and presence of BSA. Inset, Eadie-Hofstee plots for each of the profiles are shown. In the concentration of BSA at 0%, 0.2% and 0.5%, incubations were performed across 10 to 800 μM Ib concentration range. In the concentration of BSA at 1% and 2%, incubations were performed across 10 to 2000 μM Ib concentration range. The substrate concentration ([S]) in kinetic profiles refers to the unbound Ib concentration corrected by the free fraction (*f*_u_) of Ib. Each data point represents mean ± S.D. of triplicate determinations.

As shown in Figure 2, five recombinant UGTs (UGT1A3, UGT1A8, UGT1A9, UGT2B4, UGT2B7) were most active in Ib glucuronidation, so the kinetic studies were performed in these five UGTs, in the absence or presence of BSA (0.2–2%). The curves were presented in Figure 4. The kinetic parameters in UGT1A9, UGT2B4, UGT2B7 and UGT1A3, UGT1A8 were listed in Table 3. Similar to the results in HLM, Ib glucuronidation displayed substrate inhibition kinetics on all five UGTs, and the addition of BSA did not alter the enzyme kinetic of the reaction. The kinetic constants were generating by fitting the substrate inhibition model using unbound Ib concentrations. Similar to the results in Figure 2, the metabolic capacity (*V*_max_) of Ib glucuronidation in the five tested UGTs was in the order of UGT1A3 > UGT1A8 > UGT2B4 > UGT2B7 > UGT1A9. But for the metabolic affinity, the relationship between the five is UGT1A9 > UGT2B7 > UGT1A8 > UGT1A3 > UGT2B4, while the highest *K*_m_ value corresponding to the weakest affinity. So the final metabolic activity (CL_int_) of Ib glucuronidation was in the order of UGT1A3 > UGT1A8 > UGT2B7 > UGT2B4 > UGT1A9. 

However, the addition of BSA resulted in a reduction in *K*_m_ values and an increase in CL_int_ values for all investigated UGTs. Among them, BSA (0.5–2%) could make the *K*_m_ value in five UGTs decreased by about 81.5% to 98.1%. But this effect was relatively weakened at 0.2% BSA, particularly in UGT1A9 and UGT1A8. With the increase of BSA concentration from 0.2% to 2%, the growth rate of CL_int_ in UGT2B7 and UGT1A8 was increased with the increase of BSA concentration, while in other three UGTs first increased and then decreased. For UGT2B4, 0.5% BSA was corresponding to the highest CL_int_ value. For UGT1A3 and UGT1A9, 1% BSA was corresponding to the highest CL_int_ value. Interestingly, the effect of BSA on *V*_max_ was observed to have an opposite effect in different UGTs. For UGT1A9, UGT2B4 and UGT2B7, the addition of BSA could result in an increase in *V*_max_, although the increase in *V*_max_ varied with different BSA concentrations and different UGTs. However, for UGT1A3 and UGT1A8, the addition of BSA produced a sustained decline on *V*_max_ with the increase of BSA concentration. At 2% BSA, a reduction of approximately 40.5% and 26.7% on *V*_max_ value was observed for UGT1A3 and UGT1A8, respectively.

## 3. Discussion

Ib has been shown to be a potentially effective nonpeptide AT1 receptor antagonist for antihypertensive. In vitro, a significant amount of Ib monoglucuronide has been observed in liver microsomes from human and different animal species (monkey, dog, rat, mice). In vivo, by using similar investigational method [6], the amount of Ib monoglucuronide in rat bile was detected to be very abundant, accounting for about 34.2% of the total metabolites, while no Ib monoglucuronide was detected in rat urine and a very small proportion (about 1.3%) was found in rat feces. This indicated that Ib glucuronidation might be predominantly occurred in liver, not the kidney or the intestine. In addition, due to a similar structure of Ib to that of sartans, the position to which glucuronide bind (Figure 1B) might be the same as in sartans N2-tetrazole [15]. The fragmentation pathway in reported research [6] also explained that this identification is reasonable. In this manuscript, we selected liver microsomes to investigate the enzyme kinetics of Ib glucuronidation in vitro for predicting Ib elimination in vivo. And the species differences of Ib metabolism could be further clarified by comparing the kinetic parameters of Ib glucuronidation in human, dog and rat liver microsomes. As we observed, by comparing the kinetic constants of *V*_max_ and CL_int_, the metabolic capacity of Ib glucuronidation of the three species is dog > human > rat.

A panel of 12 commercially available recombinant human UGTs was evaluated for their ability to metabolize Ib, and detectable Ib monoglucuronide was generated in almost all UGTs. However, the most abundant Ib monoglucuronide was concentrated in five UGTs, with an order of UGT1A3, UGT1A8, UGT2B4, UGT2B7 and UGT1A9. Subsequent kinetic studies also yielded a similar order in the metabolic capacity of Ib glucuronidation. Eadie-Hofstee plots showed that Ib glucuronidation exhibited substrate inhibition kinetics both in pooled HLM and in human UGTs. The affinity constants (*K*_m_) were 59.8 ± 4.6, 37.6 ± 10.6, 25.6 ± 4.3, 79.3 ± 9.5, 21.6 ± 2.3 and 17.7 ± 4.6 μM for HLM, UGT1A3, UGT1A8, UGT2B4, UGT2B7 and UGT1A9, respectively. Among them, UGT1A8 was marginally expressed in liver and mainly expressed in gastrointestinal tract [16,17,18]. The other four UGTs (UGT1A3, UGT2B4, UGT2B7 and UGT1A9) were significantly expressed in liver. It is noteworthy that the *K*_m_ values of UGT1A3 and UGT2B4 were most close to the value of HLM. A substrate that is solely metabolized by a single UGT isoform should have a similar *K*_m_ value compared with that by pooled microsomes [19,20]. It seems that two UGT isoforms, probably UGT1A3 and UGT2B4, may be primarily involved in the Ib glucuronidation in HLM. Among them, UGT1A3 showed a significant high affinity and activity (*K*_m_ = 37.6 ± 10.6 μM and CL_int_ = 4.19 ± 1.21 μL·min^−1^·mg^−1^), similar to HLM (*K*_m_ = 59.8 ± 4.6 μM and CL_int_ = 3.02 ± 0.31 μL·min^−1^·mg^−1^). UGT2B4 showed a moderate affinity but a quite weak activity (*K*_m_ = 79.3 ± 9.5 μM and CL_int_ = 0.17 ± 0.04 μL·min^−1^·mg^−1^). However, UGT2B4 has found to be the most abundant UGT isoform in human liver, its mRNA expression in human liver exceeds that of UGT1A3 about 10-fold or even 290-fold [16,17]. Thus both UGT1A3 and UGT2B4 were likely to be major contributor to Ib glucuronidation in human liver. More experiments, such as chemical inhibition study and correlation analysis, need to be conducted to further confirm the major UGT subtypes involved in Ib glucuronidation in human liver. These will be described in subsequent reports. It has been reported that UGT could be inhibited by long chain unsaturated fatty acids, including arachidonic, oleic, and linoleic acid [21]. And the addition of BSA could significantly enhance the glucuronidation activity by sequestering long chain unsaturated fatty acids in the enzyme source [11,12,13,14]. However, because of the introduction of nonspecific protein binding by albumin, the dual role of BSA is proposed: first, facilitation of the reaction via an increase in the enzyme affinity of substrate; second, weaken the reaction rate via a decrease in the free fraction of substrate [7]. Therefore, a series of BSA concentration from 0.2% to 2% were adopted to adjust the kinetic data of Ib glucuronidation in liver microsomes and recombinant UGTs. Besides, the unbound fractions (*f*_u_) of Ib in buffer containing liver microsomes, supersome preparations (recombinant UGs), BSA (0–2%) and the BSA plus microsomes or supersomes were determined by ultrafiltration method. However, the unbound fraction (*f*_u_) of Ib in the enzyme source/BSA mixture was continuously decreased with the increase of BSA concentration from 0.2% to 2%. Therefore, all kinetic constants of Ib in liver microsomes and recombinant UGTs were generated by fitting with the unbound Ib concentration corrected by the *f*_u_ of Ib. The results showed that the presence of BSA could significantly reduce the *K*_m_ value and increase the CL_int_ value, both in liver microsomes and in UGTs. In addition, it was surprised to find an opposite effect of BSA on *V*_max_ value of Ib glucuronidation in different enzyme source. For RLM, DLM, HLM, UGT1A9, UGT2B4 and UGT2B7, the addition of BSA resulted in an increase in *V*_max_. But, for UGT1A3 and UGT1A8, the addition of BSA produced a sustained decline on *V*_max_ with the increase of BSA concentration. In addition, the growth rate of *V*_max_ in microsomes and UGTs all increased first and then decreased, and the highest *V*_max_ value was obtained corresponding to a relatively low concentration of BSA. However, a significant inhibitory effect on the growth of *V*_max_ could be found at 2% BSA, indicating that a high BSA concentration would weaken the positive effects of BSA itself. This negative effect at high concentrations of BSA may not be completely explained by nonspecific protein binding introduced by BSA, because the unbound Ib concentration was selected to fitting enzyme kinetics in this manuscript.

Interest continues to grow in the use of data from in vitro kinetic studies to predict in vivo hepatic clearance (CL_H_), extraction ratio, and drug-drug interaction potential using in vitro-in vivo extrapolation (IV-IVE) approaches [22,23,24,25]. Overestimation of in vitro kinetic parameters contribute to the underprediction of the in vivo kinetic parameters. Thus, its accurate estimation is therapeutically important. Recent studies from this and other laboratories have demonstrated that the predictivity of IV-IVE for substrates (and inhibitors) of several P450 and UGT enzymes is improved significantly when in vitro kinetic parameters are generated from incubations performed in the presence of bovine serum albumin (BSA). For example, Ludden et al. found that the addition of albumin to incubation media for slices or microsome experiments may yield *K*_m_ estimates for phenytoin metabolism that are more representative of in vivo values [26]. Therefore, further investigations of the influence of experimental conditions and the choice of in vitro preparations are needed to increase the predictive potential of quantitative drug metabolism data obtained in vitro. This is why our article is devoted to studying the effect of BSA on Ib glucuronidation. Atypical kinetics of substrate inhibition has been observed for Ib glucuronidation both in liver microsomes and recombinant UGTs, and the addition of BSA did not alter the enzyme kinetic of the reaction. No visible drug precipitation was found in the aqueous buffer at high concentration of Ib, and the Ib concentration was also found to be acceptable after quantitative determination, indicating that Ib was soluble at the high concentrations they used for the kinetic studies. Although the mechanism of substrate inhibition has yet to be fully determined, it has been described by a two-site model in which one binding site is productive, whereas the other site is inhibitory and operable at high substrate concentrations, resulting in decreased velocity with increasing concentrations [27]. *K*_m_ and *K*_i_ represent the affinity constants of the substrate and the two different binding sites of the enzyme, respectively. The smaller the value, the stronger the effect, vice versa. In this manuscript, for Ib glucuronidation, *K*_i_ value was much higher than *K*_m_ value both in microsomes and recombinants, the former was ten times or even hundred times to the latter. Thus, in practice, the presence of substrate inhibition in Ib glucuronidation is less likely in vivo. If the concentration of Ib in vivo accumulates to produce a substrate inhibitory effect in glucuronidation, it will cause a vicious cycle.

## 4. Materials and Methods

### 4.1. Chemicals and Materials

Ib, Ib glucuronide and irbesartan (internal standard, IS) were supplied by the Department of Medicinal Chemistry of China Pharmaceutical University, Nanjing, China. UPDGA (63700-19-6), d-saccharic acid 1,4-lactone (61278-30-6), alamethicin (27061-78-5), BSA (9048-46-8), NADP (53-59-8), G-6-P (3671-99-6), and PDH (9001-40-5) were purchased from Sigma-Aldrich (St. Louis, MO, USA). Tris-Base were purchased from Biosharp (Seoul, South Korea). 

Mixed Gender Pooled Human Liver Microsomes (HLM, from 25 subjects of 25–89 years age), Mixed Gender Pooled Cynomolgus monkey Liver Microsomes (MKLM, from 14 subjects of 4–5 years age), Mixed Gender Pooled Beagle Dog Liver Microsomes (DLM, from 7 subjects of 11 weeks age), Mixed Gender Pooled Sprague-Dawley Rat Liver Microsomes (RLM, from 100 subjects of 2–3 months age) and Mixed Gender Pooled CD1 Mice Liver Microsomes (MLM, from 500 subjects of 5–8 weeks age) were purchased from the Research Institute for Liver Diseases (Shanghai) Co. Ltd. (Shanghai, China). Supersome preparations containing recombinant UGT1A1, UGT1A3, UGT1A4, UGT1A6, UGT1A7, UGT1A8, UGT1A9, UGT1A10, UGT2B4, UGT2B7, UGT2B15 or UGT2B17 (expressed in baculovirus-infected insect cells) and UGT Inset Cell Control Supersomes^TM^ were purchased from BD Biosciences (San Jose, CA, USA). All other chemicals and solvents used were of the highest quality and analytical grade or higher.

### 4.2. LC-MS/MS Assay

The drugs were analyzed with a LC-MS/MS system fitted with SPD-20A UFLC system (Shimadzu, Tokyo, Japan) coupled to an API 4000 tandem mass spectrometer (Applied Biosciences, Foster City, CA, USA) equipped with an electrospray ionization interface. Separation of analytes was achieved on a Hypersil BDS C18 column (100 × 2.1 mm, 2.4 μm; Thermo Scientific, Waltham, MA, USA) at a flow rate of 0.10 mL/min. The mobile phase consisted of aqueous 0.02% ammonia with 1mM ammonium acetate (A) and acetonitrile (B). The following gradient elution was applied: 0–1.5 min, 15% B; 1.5–3.0 min, increase B to 50%; 3.0–4.0 min, decrease B to 40%; 4.0–8.0 min, decrease B to 30%; 8.0–9.0 min, decrease B to 15% (for a run total time of 13.0 min).

The mass spectrometer was optimized in the negative mode with ion spray voltage −4500 V, TurboSpray temperature 500 ℃, ion Source Gas 1 (N_2_) 35 Arb, Ion Source Gas 2 (N_2_) 40 Arb, Curtain Gas (N_2_) 30 Arb, Collision Gas (N_2_) 10 Arb, Entrance potential 10 V and Collision cell exit potential 12 V. Quantification was obtained using the multiple reaction monitoring (MRM) acquisition mode by monitoring the precursor ion to product ion transitions of *m*/*z* 507.0 → 284.0 at 9.10 min for Ib and *m*/*z* 427.0 → 193.1 at 8.77 min for IS, while using the neutral loss (NL) acquisition mode by monitoring the precursor ion to neutral ion transition of *m*/*z* 683.2 → 176.0 for Ib glucuronide. The acquisition and analysis of the data were performed using AB SCIEXLC/MS Analyst software, version 1.5.1.2.3 (SCIEX, Foster City, CA, USA).

### 4.3. Enzyme Incubation

Incubation mixtures contained pooled liver microsomes from human (HLM) or different animal species (monkey/MKLM, dog/DLM, rat/RLM, mice/MLM) at 1.0 mg/mL and 50 μM Ib in 100 mM potassium phosphate buffer (pH 7.4) containing 10 mM MgCl_2_. After treating with 50 μg/mg alamethicin at 4 °C for 20 min, the reactions were initiated by the addition of a NADPH-generating system (1.0 mM NADP, 10 mM G-6-P, and 1U/mL PDH) and a UDPGA-supplying system (5 mM UDPGA and 5 mM d-saccharic acid 1,4-lactone). The reaction mixtures (in 200 μL) were incubated at 37 °C for 60 min. The reaction was terminated by addition of 600 μL of acetonitrile containing 20 ng/mL IS. After vortex and centrifugation, the supernatant was submitted to HPLC-diode array detection-MS [6] and HPLC-MS/MS for Ib metabolites identification and quantification.

### 4.4. Glucuronidation in Microsomes

For optimization of the incubation conditions, the linearity of metabolite formation with time (10–90 min) and protein (0.05–1.00 mg/mL) in HLM and with time (10–60 min) and protein (0.025–0.5 mg/mL) in DLM and RLM was evaluated in advance (see Figure S1). All incubation mixtures contained 0.5 mg/mL microsomes treated with 50 μg/mg alamethicin at 4 °C for 20 min, 10 mM MgCl_2_, 5 mM d-saccharic acid 1,4-lactone, BSA (0–2%) and various concentrations of Ib in 200 μL of 50 mM Tris-HCl buffer (pH 7.5). The test concentration of Ib in the reaction was 10–800 μM at low concentration of BSA (0%, 0.2% 0.5%) and 10–2000 μM at high concentration of BSA (1%, 2%), respectively. Drug was dissolved in acetonitrile and the final concentration of acetonitrile in the reaction was 1% (*v*/*v*). The reactions were initiated by the addition of 5 mM UDPGA, incubated at 37 °C for 30 min, and then terminated by adding 600 μL of ice-cold acetonitrile containing 20 ng/mL IS. The mixtures were vortexed thoroughly and centrifugated (20,879× *g* at 4 °C for 10 min) to obtain the supernatants, of which 5 μL was subjected to analysis. Incubations without UDPGA served as negative controls.

### 4.5. Glucuronidation by Recombinant UGTs

A panel of twelve recombinant human UGTs (UGT1A1, UGT1A3, UGT1A4, UGT1A6, UGT1A7, UGT1A8, UGT1A9, UGT1A10, UGT2B4, UGT2B7, UGT2B15 and UGT2B17) was used to screen for the glucuronidation of Ib at 20 μM. Incubation conditions were similar to those of microsomes except that the protein concentration was 0.2 mg/mL. Kinetic studies for five main UGTs (UGT1A3, UGT2B4, UGT2B7, UGT1A9, and UGT1A8) were conducted with protein concentration of 0.1 mg/mL and incubation time of 30 min. The linearity of metabolite formation with time and protein in recombinant UGTs was also evaluated in advance (see Figure S2). The ranges of Ib concentrations used to obtain kinetic profiles were 10–800 μM at low concentration of BSA (0%, 0.2% 0.5%) and 10–2000 μM at high concentration of BSA (1%, 2%), respectively.

### 4.6. Protein Binding Assay of Ib

The free fraction of Ib in buffer containing liver microsomes (HLM, DLM, RLM), supersome preparations (recombinant UGT1A3, UGT1A8, UGT1A9, UGT2B4, UGT2B7), BSA (0–2%) and the BSA plus microsomes or supersomes was determined by ultrafiltration method similar to Manevski [14]. Triplicate samples of Ib were prepared in Tris-HCl buffer (pH 7.5) containing 10 mM MgCl_2_, 5 mM UDPGA, and microsomal preparation or supersome preparation in the presence or absence of BSA. Each sample (0.5 mL) was placed in a Microcon YM-10kD centrifugal filter unit (Millipore Corp., Bedford, MA, USA) following incubation (37 °C for 30 min) and centrifuged at 3000× *g* for 3 min. Control samples without microsomes or BSA were incubated simultaneously. To 50 μL of the filtrate was added 150 μL of acetonitrile containing 20 ng/mL IS. Samples were subsequently centrifuged at 20,879× *g* (Thermo Sovall Biofuge Stratos, Osterode, Germany) for 10 min to remove protein precipitates. The products in supernatant are analyzed by liquid chromatography-tandem mass spectrometry (LC-MS/MS).

### 4.7. Enzyme Kinetic Data Analysis

First, data were transformed, and Eadie-Hofstee curves were plotted, which help to identify kinetic models [28]. The apparent enzyme kinetic parameters were determined by fitting the reaction velocities versus unbound substrate concentrations present in incubations to a substrate inhibition model (Equation 1) using GraphPad Prism 6 (GraphPad Software Inc., demo, San Diego, CA, USA). CL_int_ values for Ib glucuronidation by microsomes and recombinant UGTs were determined as *V*_max_/*K*_m_.

Substrate Inhibition Model:

*v* = (*V*_max_ × [*S*])/{*K_m_* + [*S*] × (1 + [*S*]/*K_i_*)} (1)

where *v* is the rate of metabolite formation, [*S*] is the substrate concentration, *V*_max_ is the maximal velocity, *K*_m_ is the Michaelis-Menten constant (substrate concentration at 0.5 *V*_max_), *K*_i_ is the constant describing the inhibitory effect of the substrate.

## 5. Conclusions

In conclusion, this study has demonstrated that Ib monoglucuronide is the main metabolite of Ib in the liver metabolism. Ib glucuronidation both in liver microsomes and in recombinant UGTs exhibited substrate inhibition kinetics. The addition of BSA do not alter the enzyme kinetics of Ib glucuronidation, but significantly altered enzyme kinetic parameters resulting in a reduction in *K*_m_ value and an increase in CL_int_ value. However, high concentrations of BSA could attenuate this positive effect on enzyme affinity and activity. Besides, the effect of BSA on the *V*_max_ of Ib glucuronidation was opposite in different enzyme sources.

## Figures and Tables

**Table 1:** The unbound fraction (*f*_u_) of Ib in the presence of BSA. (*n* = 3, mean ± S.D.).

Ib, μM	0.2% BSA	0.5% BSA	1% BSA	2% BSA
10	0.063 ± 0.005	0.041 ± 0.003	0.022 ± 0.002	0.016 ± 0.001
20	0.114 ± 0.010	0.043 ± 0.004	0.024 ± 0.002	0.020 ± 0.001
50	0.169 ± 0.013	0.063 ± 0.005	0.026 ± 0.002	0.021 ± 0.002
100	0.339 ± 0.033	0.084 ± 0.009	0.053 ± 0.004	0.031 ± 0.002
200	0.428 ± 0.047	0.105 ± 0.010	0.095 ± 0.007	0.035 ± 0.003
400	0.559 ± 0.038	0.170 ± 0.017	0.112 ± 0.009	0.043 ± 0.005
600	0.574 ± 0.051	0.358 ± 0.028	0.117 ± 0.010	0.066 ± 0.007
800	0.577 ± 0.040	0.434 ± 0.034	0.150 ± 0.012	0.153 ± 0.008
2000	0.613 ± 0.079	0.495 ± 0.039	0.192 ± 0.015	0.183 ± 0.010

**Table 2:** Kinetic parameters of Ib glucuronidation in liver microsomes in the absence and presence of BSA. (*n* = 3, mean ± S.D.).

Enzyme Source	Parameter	No BSA	0.2% BSA	0.5% BSA	1% BSA	2% BSA
HLM	*K*_m_ (μM)	59.8 ± 4.6	5.55 ± 0.95	5.80 ± 1.80	6.10 ± 1.10	5.99 ± 0.93
	*V*_max_ (pmol/min/mg)	179.8 ± 6.7	316.3 ± 33.6	356.8 ± 37.2	389.5 ± 85.3	357.3 ± 34.4
	*K*_i_ (μM)	455.5 ± 59.9	300.3 ± 86.5	350.0 ± 106.6	200.3 ± 92.4	354.1 ± 106.5
	CL_int_ (μL/min/mg)	3.02 ± 0.31	57.8 ± 9.6	64.4 ± 14.3	66.0 ± 20.6	61.2 ± 15.4
DLM	*K*_m_ (μM)	9.00 ± 2.10	1.48 ± 0.33	1.44 ± 0.20	1.40 ± 0.19	1.11 ± 0.23
	*V*_max_ (pmol/min/mg)	890.0 ± 44.0	1712.0 ± 308.1	1533.0 ± 122.81	1492.0 ± 71.4	1182.0 ± 65.4
	*K*_i_ (μM)	233.6 ± 54.0	199.0 ± 59.5	89.7 ± 19.9	74.1 ± 10.0	226.4 ± 20.0
	CL_int_ (μL/min/mg)	101.9 ± 19.4	1164.5 ± 52.8	1086.6 ± 38.5	1083.7 ± 99.9	1088.2 ± 70.2
RLM	*K*_m_ (μM)	13.0 ± 3.1	1.83 ± 0.78	2.00 ± 0.12	1.92 ± 0.51	1.80 ± 0.40
	*V*_max_ (pmol/min/mg)	28.5 ± 2.4	41.5 ± 2.3	57.2 ± 10.3	37.5 ± 2.0	36.5 ± 3.7
	*K*_i_ (μM)	332.9 ± 50.3	187.5 ± 34.3	176.2 ± 84.6	270.0 ± 53.9	263.7 ± 58.0
	CL_int_ (μL/min/mg)	2.25 ± 0.36	25.6 ± 5.6	28.8 ± 6. 9	20.7 ± 6.8	20.7 ± 2.6

All kinetic constants were generating by fitting the substrate inhibition model using unbound Ib concentrations.

**Table 3:** Kinetic parameters of Ib glucuronidation in UGT1A9, 2B4, 2B7 and UGT1A3, 1A8 in the absence and presence of BSA. (*n* = 3, mean ± S.D.).

Enzyme Source	Parameter	No BSA	0.2% BSA	0.5% BSA	1% BSA	2% BSA
UGT1A9	*K*_m_ (μM)	17.7 ± 4.6	20.8 ± 4.3	1.61 ± 0.52	1.23 ± 0.42	1.06 ± 0.21
	*V*_max_ (pmol/min/mg)	1.79 ± 0.43	10.1 ± 4.5	6.39 ± 0.93	5.73 ± 0.70	3.85 ± 0.22
	*K*_i_ (μM)	508.8 ± 134.4	236.1 ± 46.1	251.7 ± 38.7	96.6 ± 36.4	185.2 ± 52.8
	CL_int_ (μL/min/mg)	0.10 ± 0.03	0.49 ± 0.19	4.14 ± 0.80	5.52 ± 1.48	3.75 ± 0.92
UGT2B4	*K*_m_ (μM)	79.3 ± 9.5	7.40 ± 2.40	1.65 ± 0.28	3.70 ± 0.96	3.65 ± 0.70
	*V*_max_ (pmol/min/mg)	13.6 ± 2.0	33.2 ± 3.4	43.8 ± 1.9	28.7 ± 2.2	21.5 ± 1.3
	*K*_i_ (μM)	682.7 ± 148.7	844.0 ± 101.5	352.6 ± 60.6	321.0 ± 106.3	1104.0 ± 236.5
	CL_int_ (μL/min/mg)	0.17 ± 0.04	4.95 ± 1.18	27.1 ± 5.1	8.20 ± 2.71	6.09 ± 1.55
UGT2B7	*K*_m_ (μM)	21.6 ± 2.3	6.00 ± 1.31	2.51 ± 0.65	0.99 ± 0.30	0.56 ± 0.11
	*V*_max_ (pmol/min/mg)	4.07 ± 0.50	9.63 ± 1.81	7.10 ± 1.04	5.95 ± 0.72	4.31 ± 0.20
	*K*_i_ (μM)	478.4 ± 169.3	304.0 ± 153.9	415.6 ± 83.6	172.0 ± 56.5	146.6 ± 31.4
	CL_int_ (μL/min/mg)	0.20 ± 0.04	1.52 ± 0.18	2.66 ± 0.68	6.25 ± 1.22	8.92 ± 0.78
UGT1A3	*K*_m_ (μM)	37.6 ± 10.6	4.35 ± 1.37	1.40 ± 0.21	0.70 ± 0.16	0.75 ± 0.10
	*V*_max_ (pmol/min/mg)	146.8 ± 21.2	124.3 ± 13.5	107.8 ± 6.5	100.1 ± 14.5	87.4 ± 2.7
	*K*_i_ (μM)	862.7 ± 265.8	607.4 ± 44.6	499.6 ± 85.9	452.7 ± 154.3	816.5 ± 189.0
	CL_int_ (μL/min/mg)	4.19 ± 1.21	30.2 ± 7.8	77.9 ± 9.6	144.9 ± 22.7	118.3 ± 19.5
UGT1A8	*K*_m_ (μM)	25.6 ± 4.3	16.0 ± 5.7	4.73 ± 1.05	2.13 ± 0.34	1.29 ± 0.29
	*V*_max_ (pmol/min/mg)	24.3 ± 3.5	21.9 ± 1.2	19.3 ± 4.9	18.4 ± 3.6	17.8 ± 1.1
	*K*_i_ (μM)	606.4 ± 156.8	357.5 ± 91.1	209.6 ± 15.2	218.4 ± 83.7	179.9 ± 43.1
	CL_int_ (μL/min/mg)	0.96 ± 0.14	1.52 ± 0.65	4.16 ± 1.10	8.68 ± 1.48	14.21 ± 2.74

All kinetic constants were generating by fitting the substrate inhibition model using unbound Ib concentrations.

## Supplementary Materials

The following are available online at http://www.mdpi.com/1420-3049/23/3/688/s1, Figure S1: Correlation of metabolite formation with incubation time in (**a**) human liver microsomes/HLM; (**b**) dog liver microsomes/DLM; (**c**) rat liver microsomes/RLM; (**d**) recombinant human UGT1A3 and (**e**) recombinant human UGT2B4 (n = 3). Figure S2: Correlation of metabolite formation with protein concentration in (**a**) human liver microsomes/HLM; (**b**) dog liver microsomes/DLM; (**c**) rat liver microsomes/RLM; (**d**) recombinant human UGT1A3 and (**e**) recombinant human UGT2B4 (n = 3).

## Abbreviations

Ib	5-*n*-butyl-4-{4-[2-(1*H*-tetrazole-5-yl)-1*H*-pyrrol-1-yl]phenylmethyl}-2,4-dihydro-2-(2,6-dichloro- phenyl)-3*H*-1,2,4-triazol-3-one
AT1	angiotensin type 1
BSA	bovine serum albumin
IS	internal standard
UPDGA	uridine 5’-diphosphoglucuronic acid
NADP	β-nicotinamide adenine dinucleotide phosphate
G-6-P	d-glucose 6-phosphate
PDH	glucose-6-phosphate dehydrogenase
HLM	human liver microsomes
MKLM	monkey liver microsomes
DLM	dog liver microsomes
RLM	rat liver microsomes
MLM	mouse liver microsomes
UGT	UDP-glucuronosyltransferases
CYP	cytochrome P450
LC-MS/MS	liquid chromatography-tandem mass spectrometry
DDI	drug-drug interactions
IVIVE	in vitro-in vivo extrapolation
