# Supplementary material for: Atypical Kinetics and Albumin Effect of Glucuronidation of 5-n-Butyl-4-{4-[2-(1H-tetrazole-5- yl)-1H-pyrrol-1-yl]phenylmethyl}-2,4-dihydro-2-(2,6- dichlorophenyl)-3H-1,2,4-triazol-3-one, a Novel Nonpeptide Angiotensin Type 1 Receptor Antagonist, in Liver Microsomes and UDP-Glucuronosyl-transferase
Source: Molecules. 2018 Mar 19;23(3):688. doi: 10.3390/molecules23030688 (PMC6017351; doi:10.3390/molecules23030688)
Supplement: Supplementary file 1 [file molecules-23-00688-s001.pdf]

Supplementary data

Atypical Kinetics and Albumin Effect of Glucuronidation of 5-*n*-Butyl-4-{4-[2-(1*H*-tetrazole-5-yl)-1*H*-pyrrol-1-yl]phenylmethyl}-2,4-dihydro-2-(2,6-dichlorophenyl)-3*H*-1,2,4-triazol-3-one, a Novel Nonpeptide Angiotensin Type 1 Receptor Antagonist, in Liver Microsomes and UDP-Glucuronosyl-transferase

Ying Peng, Xueyuan Zhang, Yinci Zhu, Hui Wu, Shiyin Gu, Qingqing Chang, Yi Zhou, Guangji Wang * and Jianguo Sun *

Key Lab of Drug Metabolism and Pharmacokinetics, State Key Laboratory of Natural Medicines, Pharmaceutical University China, Nanjing 210009, Jiangsu, China

* Correspondence: guangjiwang@hotmail.com (G.W.); jgsun@cpu.edu.cn (J.S.); Tel.: +86-25-8327-1128 (G.W.); +86-25-8327-1176 (J.S.); Fax: +86-25-8327-1060 (G.W. & J.S.)

Optimization of reaction time and protein concentration of Ib glucuronidation in microsome and recombinant UGTs

1. Optimization of the reaction time

For optimization of the incubation time in microsomes, the formation of Ib monoglucuronide with time (10–90 min) in human liver microsomes (HLM) and with time (10-60 min) in dog liver microsomes (DLM) and rat liver microsomes (RLM) was evaluated. All incubation mixtures contained 0.2 mg/ml microsomes treated with 50 μg/mg alamethicin at 4 °C for 20 min, 10 mM $MgCl_2$, 5 mM D-saccharic acid 1,4-lactone and 10 μM Ib in 200 μL of 50 mM Tris-HCl buffer (pH 7.5). The reactions were initiated by the addition of 5 mM UDPGA, incubated at 37 °C for investigated times, and then terminated by adding 600 μL of ice-cold acetonitrile containing 20 ng/mL IS. The mixtures were vortexed thoroughly and centrifugated (20,879× *g* at 4°C for 10 min) to obtain the supernatants, of which 5 μL was subjected to analysis.

For optimization of the incubation time in recombinant UGTs, the formation of Ib monoglucuronide with time (10–60 min) in UGT1A3 and UGT2B4 was evaluated. Incubation conditions were similar to those of microsomes except that the protein concentration was 0.1 mg/ml and the investigated concentration of Ib was 20 μM.

A curve was constructed by plotting the metabolite formation (the concentration of Ib monoglucuronide) versus nominated incubation time, and the linearity between concentration and time was evaluated. The optimal reaction time was selected according to four points: a reduction of no more than 10% of Ib, a linear increase in metabolite production over time, a maximum possible formation of metabolite in the linear range and operability. As shown in Supplementary Figure 1, the best incubation time in the microsomes of three different species can be unified for 20 min. For recombinant UGTs, the choice of 30 min may be suitable for all investigated UGTs (UGT1A3, UGT1A8, UGT1A9, UGT2B4, UGT2B7) in the main text of this manuscript.

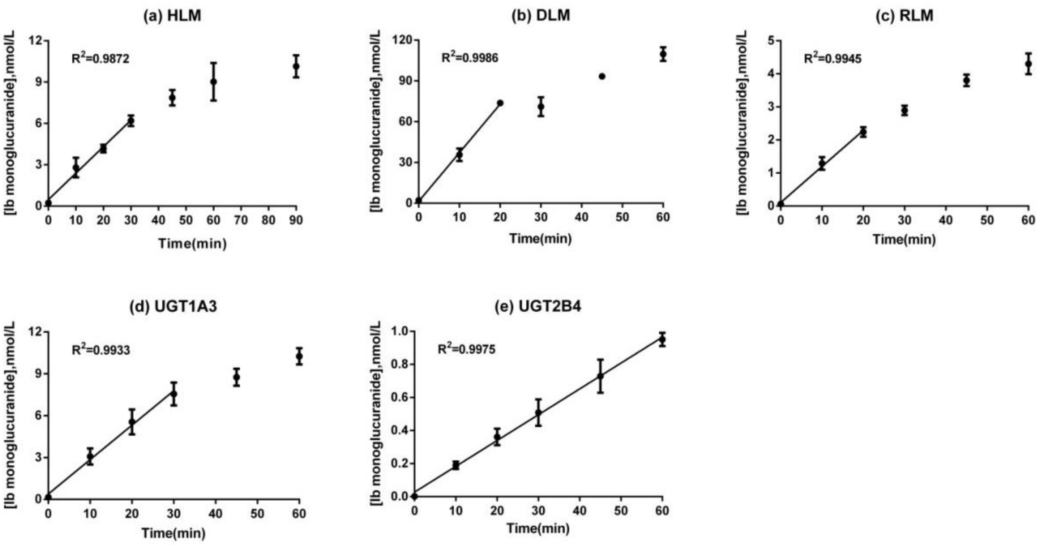

Figure S1. Correlation of metabolite formation with incubation time in (**a**) human liver microsomes/HLM, (**b**) dog liver microsomes/DLM, (**c**) rat liver microsomes/RLM, (**d**) recombinant human UGT1A3 and (**e**) recombinant human UGT2B4 (n=3).

2. Optimization of Protein Concentration

For optimization of the protein concentration in microsomes, the formation of Ib monoglucuronide with protein (0.05–1.0 mg/mL) in HLM and with protein (0.025–0.5 mg/mL) in DLM and RLM was evaluated. For optimization of the protein concentration in recombinant UGTs, the formation of Ib monoglucuronide with protein (0.025–0.5 mg/mL) in UGT1A3 and UGT2B4 was evaluated. Incubation conditions were similar to those described in above section 1 except that the incubation time was fixed at 20 min on microsomes and 30 min on recombinant UGTs.

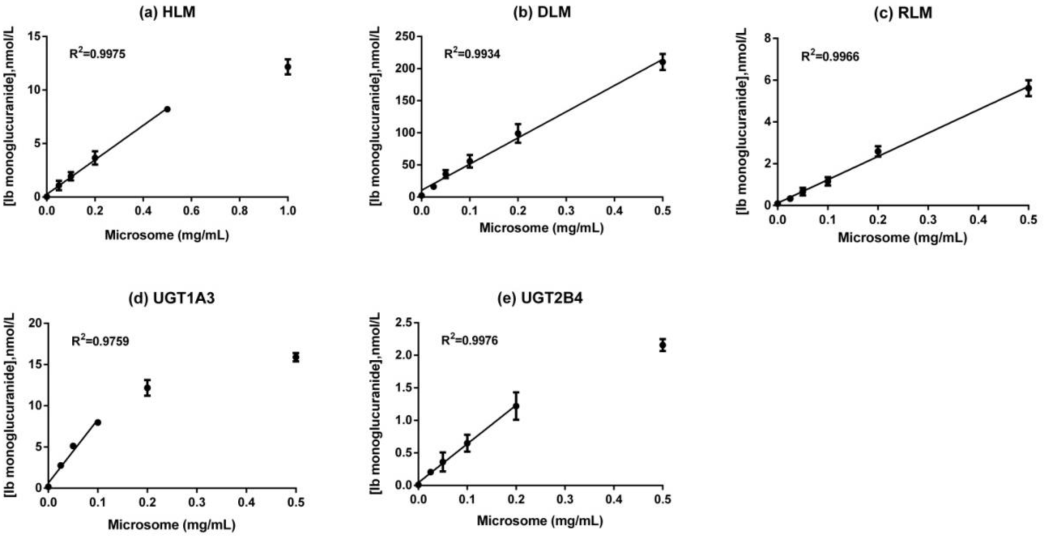

Figure S2. Correlation of metabolite formation with protein concentration in (**a**) human liver microsomes/HLM, (**b**) dog liver microsomes/DLM, (**c**) rat liver microsomes / RLM, (**d**) recombinant human UGT1A3 and (**e**) recombinant human UGT2B4 (n=3).

A curve was constructed by plotting the metabolite formation (the concentration of Ib monoglucuronide) versus nominated protein concentration, and the linearity between metabolite concentration and protein concentration was evaluated. The optimal protein concentration was selected according to similar four points as the reaction time. As shown in Supplementary Figure 2,

the best protein concentraion in the microsomes of three different species can be unified for 0.5 mg/mL. For recombinant UGTs, the choice of 0.1 mg/mL may be suitable for all investigated UGTs (UGT1A3, UGT1A8, UGT1A9, UGT2B4, UGT2B7) in the main text of this manuscript.
